# Artificial Intelligence for RECIST-Based Radiologic Treatment Response Assessment in Solid Tumors: A Systematic Review of Imaging- and Report-Derived Approaches

**DOI:** 10.3390/cancers18050808

**Published:** 2026-03-02

**Authors:** Agnieszka Leszczyńska, Michał Seweryn, Rafał Obuchowicz, Michał Strzelecki, Adam Piórkowski, Paweł Michał Potocki

**Affiliations:** 1 EconMed Europe, Młyńska 9/4, 31-469 Krakow, Poland; agnieszka.leszczynska@autograf.pl (A.L.); misewer@gmail.com (M.S.);; 2 Faculty of Health Sciences, Andrzej Frycz Modrzewski Krakow University, Gustawa Herlinga-Grudzińskiego 1, 30-705 Krakow, Poland; 3 Lux Med Ltd., 02-678 Warsaw, Poland; r.obuchowicz@gmail.com; 4 Department of Diagnostic Imaging, Jagiellonian University Medical College, 31-008 Krakow, Poland; 5 Institute of Electronics, Lodz University of Technology, 93-590 Lodz, Poland; 6 Department of Biocybernetics and Biomedical Engineering, AGH University of Kraków, 30-059 Krakow, Poland; 7 Department of Oncology, Jagiellonian University Medical College, 31-008 Krakow, Poland

**Keywords:** artificial intelligence, deep learning, LLM, natural language processing, precision oncology, radiology report, RECIST 1.1, treatment response assessment, solid tumors, cross-sectional imaging

## Abstract

Background/Objectives: To systematically review and critically appraise AI methods for RECIST-based radiologic treatment response assessment in solid tumors, comparing image-derived and report-derived approaches and summarizing their performance, agreement with reference standards, and validation quality. Methods: This systematic review followed PRISMA guidelines. We searched Embase, MEDLINE, Web of Science, Scopus, and the Cochrane Library on 6 December 2025. We included English-language original studies (2015–2025) in solid tumors where AI directly assigned RECIST response categories and was validated against a reference standard; studies without RECIST-based response endpoints or non–solid tumor populations were excluded. We distinguished image-based techniques that assign RECIST categories from direct analysis of imaging data from report-based techniques that infer RECIST categories from radiology reports using natural language processing. Results: Evidence remains sparse; we identified four eligible studies (two image-based and two report-based). DeepSeek-V3-0324 and GatorTron, both report-based approaches, achieved high accuracy (96.5% and 89%, respectively) in treatment response evaluation, with DeepSeek demonstrating higher expert agreement (κ 0.85–0.90). The nnU-Net and 3D U-Net pipelines, both image-based, showed high segmentation performance (DSC 0.85, VS 0.89) and treatment response classification accuracy of 0.77 for R1, with moderate agreement with the manual reference (κ = 0.60); nnU-Net also achieved moderate to almost perfect agreement (Cohen’s κ 0.67–0.81) in RECIST 1.1 measurements. Conclusions: AI-based RECIST-oriented response assessment is feasible and potentially beneficial for standardization, efficiency, and scalability, but current evidence is limited and heterogeneous, requiring larger multi-center studies with rigorous external validation before clinical adoption. Key limitations include data source variability, reference standard inconsistencies, and lack of robust external validation.

## Simple Summary

Response Evaluation Criteria in Solid Tumors (RECIST) are widely used to determine whether cancer treatments are working, but the manual assessment performed by radiologists is time-consuming and can vary between evaluators. Artificial intelligence (AI) offers the potential to make RECIST assessments faster, more consistent, and easier to scale. In this systematic review, we examined AI methods developed to automatically determine treatment response in patients with solid tumors. We analyzed approaches based on medical images as well as those interpreting radiology reports. Although only a small number of studies met our criteria, the results show that AI can accurately assign RECIST response categories and may improve efficiency. However, the existing evidence is still limited, varies widely in methodology, and often lacks strong external validation. Larger, multi-center studies are needed to confirm the reliability of these tools before they can be used routinely in clinical practice.

## 1. Introduction

Assessment of treatment response in solid tumors is a key component of therapeutic management, eligibility for subsequent lines of therapy, and evaluation of efficacy in clinical trials [1]. The most commonly used standardized approach is Response Evaluation Criteria in Solid Tumors (RECIST), which compares lesion dimensions across consecutive imaging examinations and assigning response categories: complete response (CR), partial response (PR), stable disease (SD) or progressive disease (PD) [2]. Despite its widespread use, RECIST assessment is time-consuming and susceptible to inter-observer variability, for example, due to target lesion selection, measurement technique, and interpretation of progression [3,4]. In addition, because RECIST relies on fixed thresholds, relatively small measurement differences—particularly for lesions near decision cut-offs—may result in different response classifications and, potentially, inconsistent clinical decisions [3,5]. In light of the limitations of traditional response assessment, artificial intelligence (AI) offers a promising approach to improving the consistency and efficiency of RECIST-based evaluation. Machine learning-based (ML) image analysis may help integrate imaging biomarkers, reduce subjectivity in measurements, and improve the reproducibility of response assessments [6,7]. In this context, AI is being explored to automate key steps in the RECIST workflow, including lesion detection and segmentation, extraction of RECIST-compliant measurements, longitudinal lesion tracking across timepoints, and response classification. In practice, these tools may pre-populate lesion annotations and RECIST measurement tables for radiologist verification, link lesions across baseline and follow-up scans to streamline longitudinal assessment, and flag borderline changes near RECIST cut-offs for focused review. Overall, the goal is to enhance reproducibility while simultaneously reducing workload and minimizing reader variability, which is especially relevant for measurement-based standards such as RECIST.

Solid tumors are difficult to measure due to their irregular shape, varying location, and reliance on imaging techniques with different resolutions, which complicates accurate size determination and treatment response assessment [8]. RECIST was developed to standardize imaging-based response assessment; however, it remains susceptible to measurement errors and reader-dependent choices. In this context, artificial intelligence can serve as a tool to support diagnostics and treatment response evaluation. AI excels at recognizing complex patterns in images, and this process is objective and repeatable, also enabling the rapid extraction of progression signals from real-world data without the need for image segmentation [9]. Additionally, the results provided by AI serve as an objective “benchmark” in comparative tasks, helping to identify areas of greatest discrepancy and potential sources of errors or oversights among radiologists assessing treatment response based on, for example, CT results [10]. This thus complements clinical decision-making and acts as a means of evaluating (auditing) the work of radiologists [9].

In clinical workflows, RECIST automation could be implemented as decision support during reporting, for example, by automatically pre-populating target-lesion measurements and longitudinal comparisons and flagging potential progression events (e.g., new lesions or non-target progression) for radiologist confirmation [4]. In clinical trials, such tools could support blinded independent central review by standardizing measurements and reducing manual effort while preserving auditable outputs. By contrast, report-based NLP systems primarily enable retrospective capture of responses from routine documentation for registries or real-world evidence analyses, rather than replacing measurement steps on images [11,12].

Nowadays, two main directions of AI application in analyzing the progression of malignant tumors can be distinguished. The first of these, image-based approaches directly analyze imaging data, such as computed tomography (CT) or magnetic resonance imaging (MRI), to detect and/or segment lesions, derive RECIST-compliant size measurements (longest diameter for non-nodal target lesions and short-axis diameter for lymph nodes), and assign response categories at the patient level [4,7]. Their performance is influenced by imaging-related factors such as acquisition protocol, reconstruction parameters, slice thickness, contrast phase, and registration/longitudinal lesion matching; moreover, differences in target lesion selection and tracking can propagate into the final RECIST category, particularly when progression depends on new lesions or non-target disease [3]. Accordingly, image-based systems are commonly evaluated against radiologist measurements and categories (often via blinded independent central review) using both categorical agreement on RECIST classes and continuous measures of measurement error or inter-method agreement. The second, report-based approach uses natural language processing (NLP) methods—including large language models (LLMs)—to infer treatment response from radiology reports, reconstructing the RECIST category (or a close proxy) without reprocessing the raw images [11]. These methods rely on information summarized by the reporting radiologist, such as explicit RECIST terminology (e.g., “partial response”, “progression”), descriptions of size changes, statements about new lesions, or impression-level conclusions indicating stability versus progression; therefore, performance depends strongly on reporting practices (structured templates vs. free text), the consistency of RECIST language, and local documentation conventions [1,11]. A comparison of image-based and report-based approaches, together with their advantages and limitations, is presented in Table 1.

The fragmentation of the evidence base for artificial intelligence directly assigning RECIST response categories, within two conceptually distinct streams—image-based and report-based—presents certain challenges [13]. Specifically, their results, reference standards, and performance metrics are not directly comparable. As a result, the clinical and methodological implications, including the strength of validation and generalizability, are difficult to interpret based on the existing literature.

Image-based methods attempt to reproduce the RECIST process from the ground up (lesion identification, measurement, and longitudinal comparison), and therefore are benchmarked on measurement accuracy, lesion-level agreement, and reader-to-model concordance under controlled imaging conditions [14]. Report-based methods, in contrast, infer response from an already interpreted narrative and are benchmarked primarily on text classification performance (e.g., accuracy) against labels derived from reports, registries, or expert annotation [15]. Mixing these outcomes risks comparing non-equivalent endpoints—continuous measurement agreement versus categorical label extraction—and conflating errors arising from image acquisition and measurement with errors arising from documentation style and linguistic ambiguity. Moreover, the clinical meaning of “ground truth” differs: for report-based models, the target is typically the documented conclusion (what was stated), whereas for image-based models, the target is the imaging-derived assessment (what is measurable on scans, ideally under standardized review). Therefore, to maintain interpretability and avoid methodological heterogeneity in this work, the results obtained from image-based and report-based approaches will be analyzed individually in separate sections, discussing modality-specific results, biases, and applicability issues independently.

This systematic literature review (SLR) seeks to address this gap by synthesizing the two streams separately, offering a clear comparison of their tasks, reference standards, validation strategies, and sources of error relevant to clinical translation. The goal of this review is to compile and critically appraise the evidence on the use of AI for RECIST-based radiologic treatment response assessment in solid tumors, distinctly differentiating between image-derived and report-derived methods.

This review focuses on studies in which AI directly assigns response categories (CR/PR/SD/PD or equivalent) and in which outcomes were formally validated against a reference standard. Specifically, this review seeks to answer the following questions: (1) what performance is achieved and how closely results agree with the reference assessment—including measurement error and agreement metrics for image-based methods, versus classification performance for report-based methods, (2) what the quality and scope of validation (internal/external) are, and (3) which methodological limitations may affect the generalizability and clinical utility of these tools.

## 2. Materials and Methods

### 2.1. Study Design and Search Strategy

This systematic review was conducted in accordance with the Preferred Reporting Items for Systematic Reviews and Meta-Analyses (PRISMA) reporting guidelines to ensure a comprehensive and transparent approach [16]. The protocol for the systematic review of our study was not registered for PROSPERO. The search was executed on 6 December 2025 across Medline, Scopus, Web of Science, Embase, and the Cochrane Library. A strategy combining medical subject headings (MeSH) terms, Boolean logic, and pertinent keywords was used to obtain a broad yet precise collection of studies. The exact search strings for each database are provided in Table A1 (Appendix A).

### 2.2. Eligibility Criteria and Study Selection

Eligibility was limited to studies published in English between 2015 and 2025 that evaluated AI methods for RECIST-based radiologic treatment response assessment in solid tumors, in which the model directly assigned response categories (CR/PR/SD/PD or equivalent) and was formally validated against a reference standard.

Studies were excluded if they did not apply RECIST (or RECIST-equivalent categories) as the response endpoint, automated only parts of the RECIST assessment (i.e., only segmentation or diameter measurements), focused solely on detection/diagnosis or prognostic prediction without response assessment, used non-LLM methods for extraction of data from radiology reports, involved non-human or non–solid tumor studies, or were non-original publications. Additionally, studies focused solely on volumetric response, radiomics signatures, or alternative AI-derived endpoints were excluded, as these approaches, while potentially informative, are not directly translatable into RECIST 1.1 response classes and could introduce substantial clinical and methodological heterogeneity. Where studies included volumetric or radiomics analyses alongside RECIST outputs, only the RECIST-relevant results were extracted. This approach ensured the selection of studies with practical clinical relevance stemming from the application of current AI advances.

**Figure 1:**
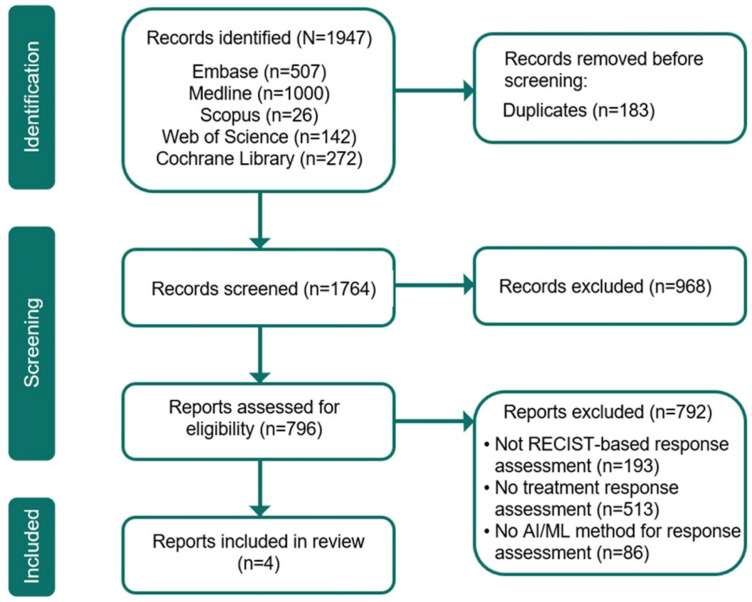
PRISMA flow chart. AI: artificial intelligence; ML: machine learning; PRISMA: Preferred Reporting Items for Systematic Reviews and Meta-Analyses; RECIST: Response Evaluation Criteria in Solid Tumors.

Study selection was performed independently by two reviewers (A.L. and M.S.) in a blinded manner. Titles and abstracts were screened first, followed by full-text review against predefined eligibility criteria. Disagreements were resolved through discussion; if consensus could not be reached, a third reviewer adjudicated. The selection process is summarized in the PRISMA flow diagram (Figure 1).

### 2.3. Data Extraction and Assessment

Extracted data included study characteristics, population and cancer type, imaging modality and timepoints, data source (images vs. radiology reports), AI application, and reference standard for RECIST response. For each study, we recorded validation strategy (internal/external), sample size, and performance metrics, prioritizing agreement with the reference standard (e.g., Cohen’s/Fleiss’ κ) and classification performance for response categories. Findings were summarized in tables, separately for image-derived and report-derived approaches.

Risk of bias and applicability were assessed independently by two reviewers using the QUADAS-2 tool [17] (see Table A2, Appendix A) and, for studies evaluating multivariable prediction models (including AI/ML/LLM approaches), the prediction model risk of bias assessment tool (PROBAST) (see Table A3, Appendix A). QUADAS-2 was used because it provides a transparent and widely accepted framework for evaluating risk of bias and concerns regarding applicability in diagnostic test accuracy studies comparing an index test with a reference assessment across four key domains: patient selection, index test, reference standard, and flow and timing. Although QUADAS-2 was not originally developed for AI-based models and does not explicitly address AI-specific threats (e.g., data leakage, iterative model/prompt optimization, or domain shift), it supported a consistent and comparable appraisal across the heterogeneous study designs included in this review. PROBAST was included to complement QUADAS-2 by addressing key sources of bias specific to prediction-model development and validation, across four domains: participants, predictors, outcome, and analysis. For QUADAS-2, signaling questions were rated as “yes,” “no,” or “unclear,” informing domain-level judgments of low, unclear, or high risk of bias; applicability concerns were assessed for the first three domains. For PROBAST, signaling questions were rated as “yes”, “probably yes”, “probably no”, “no”, or “no information”, informing domain-level and overall judgments of low, unclear, or high risk of bias, as well as concerns regarding applicability. Discrepancies between reviewers were resolved by discussion (and, where needed, consultation with a third reviewer).

## 3. Results

The initial search across the databases returned 1947 articles, with 183 duplicates eliminated. After screening the titles and abstracts, 968 articles were excluded as they did not satisfy the inclusion criteria. The full text of the remaining 796 articles was then evaluated. 792 articles were excluded because they were not RECIST-based, did not assess treatment response, or did not use AI/ML methods. Of the four included publications, two employed an image-based approach based on raw imaging data, and two used a report-based approach in which language models analyze radiology report texts (Table 2).

**Table 2:** Characteristics of research and data.

Study	Study Characteristics	Clinical Context	AI Application	Validation
Reports-based studies				
Tan et al. (2023) [18]	- retrospective - single center (Singapore; with an additional external clinical-trial cohort) - English-language - CT - solid tumors (colorectal, breast, gynecological, and lung) - *n* = 1740 patients (10,602 CT reports; 37 RECIST-annotated trial reports)	- automated assignment of treatment response categories (NED/PR/SD/PD; RECIST 1.1) - from free-text radiology reports (multiple reports, per patient) - performance evaluated by 3 clinicians using unstructured (free-text) report data	- transformer-based LLM (GatorTron; transformer encoder) - task-specific fine-tuning - standard inference after fine-tuning (no few-shot or chain-of-thought prompting) - no RAG - comparators: Bi-LSTM, CNN, and classical ML baselines - evaluation objective: comparison of GatorTron performance against DL/ML baselines on the same task	- internal: single train/development/test split, with hyperparameter tuning on the development set and final evaluation on an independent test set - external: an external clinical-trial report cohort with centrally adjudicated RECIST 1.1 assessments (clinical trial in ovarian cancer)
Yang et al. (2025) [19]	- retrospective - multi center (China, 3 sites) - Mandarin-language - MRI - NPC - *n* = 307 patients (924 reports) + an independent test set of 277 patients (831 reports)	- automated assignment of baseline TNM and of treatment response categories (CR/PR/SD/PD; RECIST 1.1) - from free text radiology reports (3 subsequent reports per patient) - performance evaluated by 4 clinicians using unstructured (free-text) report data	- transformer-based LLMs (DeepSeek-V3-0324 and GPT-4o-latest) - no task-specific fine-tuning on local data - few-shot prompting with chain-of-thought–style reasoning prompts - RAG - comparison between LLMs under the same prompting and RAG setup - assessment of LLM performance in a no-training setting (prompting + RAG)	- internal: head-to-head evaluation comparison of two LLMs on the same retrospective dataset with statistical testing; no separate model training/validation procedure - external: no independent external validation cohort; evaluation was performed across three hospitals within affiliated network (multi-site, but not externally locked/independent)
Image-based studies				
Liu et al. (2022) [20]	- retrospective - single center (China) - baseline and post-treatment MRI (3.0T; DWI-based workflow, standardized protocol) - hepatic metastases from various solid tumors - *n* = 116 patients (299 MRI scans), including training dataset of 86 patients (238 scans) and a validation dataset	- automated assignment of treatment response categories (CR/PR/SD/PD; RECIST 1.1) - automated 3D segmentation of the liver and metastases (automatic lesion selection) - performance evaluated against manual radiologist-derived RECIST 1.1 assessment on paired baseline and post-treatment scans	- deep learning 3D U-Net–based segmentation pipeline (two-stage: liver segmentation → metastasis segmentation within the liver mask) - supervised training for the segmentation tasks (liver and metastases) - sequential two-step inference with metastasis segmentation constrained by the liver mask - no RAG - manual radiologist-derived RECIST 1.1 reference standard as a comparator; additional comparison to individual radiologist readings - assess automated segmentation performance and agreement with RECIST 1.1 reference and radiologist readers	- internal: random hold-out validation within the initial cohort (*n* = 86), split 6:2:2 into train (52)/validation (17)/test (17), with performance reported for both the test set and reader comparisons - external: no independent multi-institutional external cohort; instead, an independent temporal validation cohort was used (*n* = 30, later time period) from the same overall clinical setting to assess generalizability
Dahm et al. (2024) [21]	- retrospective - multi-center (Germany/New Zealand; 3 sites), multi-observer - baseline and first post- treatment CT - metastatic melanoma (MM) - *n* = 58 patients (114 lesions) with paired baseline and first follow-up CT	- automated assignment of treatment response categories (CR/PR/SD/PD; RECIST 1.1 and additionally according to volume change using RECIST mimicking response criteria) - automated 3D segmentation of the liver and metastases (manual lesion selection) - performance evaluated against segmentation assisted radiologist-derived RECIST 1.1 assessment on paired baseline and post-treatment scans	- automatic target-lesion segmentation and longitudinal matching on follow-up CT (nnU-Net + deformable registration) - supervised training for target-lesion segmentation (nnU-Net) and algorithmic longitudinal matching via deformable registration - segmentation on serial CTs → deformable registration–based matching across timepoints → automated extraction of RECIST 1.1 diameters and volumes - no RAG - radiologist readers as the reference for standard RECIST; automated standard and volumetric RECIST assessed against reader evaluations - evaluate automated lesion segmentation, longitudinal consistency/matching, and RECIST (diameter-based) and volumetric response assessment versus radiologists	- internal: manual RECIST 1.1 measurements by three radiologists served as the reference standard; agreement with automated diameter- and volume-based outputs were evaluated using ICC and kappa - external: evaluation was conducted on a multicenter MM CT cohort.; however, an independent externally locked test set was not explicitly specified (external evaluation reported, independence unclear). The nnU-Net component was pre-trained on >16,000 lesions from multiple hospitals

Bi-LSTM: bidirectional long short-term memory; CNN: convolutional neural network; CR: complete response; CT: computed tomography; DL: deep learning; DWI: diffusion-weighted imaging; GPT: Generative Pre-trained Transformer; ICC: intraclass correlation coefficients; LLM: large language models; ML: machine learning; MM: metastatic melanoma; MRI: magnetic resonance imaging; NED: no evidence of disease; NPC: nasopharyngeal carcinoma; PD: progressive disease; PR: partial response; RAG: retrieval-augmented generation; RECIST: Response Evaluation Criteria in Solid Tumors; SD: stable disease.

Among the included studies, only Yang et al. evaluated LLM performance in a multicenter cohort, and Dahm et al. represented a multi-center, multi-observer reading study; however, both were retrospective.

Tan et al. provided an external RECIST-based validation cohort derived from an investigator-initiated clinical trial, whereas Liu et al. relied on a single-center retrospective dataset and explicitly noted the need for multi-center data to establish robustness.

Because the included studies assessed different stages of the RECIST workflow, evaluation outcomes are summarized in Table 3 by evaluation level and metric type to facilitate comparability.

### 3.1. Quality Assessment

The methodological quality of the included studies was critically appraised using the QUADAS-2 tool. Overall, the evidence base showed important limitations, with the most consistent concerns arising in the patient selection and applicability domains. In terms of risk of bias, Dahm et al.’s study was judged at low risk for patient selection due to random sampling, whereas the remaining studies had unclear-to-high risk, mainly driven by restrictive inclusion criteria and/or substantial exclusions (e.g., measurable-lesion requirements and exclusion of indeterminate cases). Risk of bias related to the index test was generally low to unclear, but was considered high in one study due to iterative prompt refinement aimed at maximizing performance, raising concerns about potential overfitting and insufficient separation between optimization and evaluation. The reference standard was judged low risk where independent expert adjudication was clearly described, but was unclear or high risk where the standard relied on heterogeneous clinical confirmation and/or report-derived labels rather than a direct imaging-based gold standard. Regarding flow and timing, most studies were rated low or unclear, reflecting incomplete reporting of patient flow and consistency of follow-up across analyzed datasets. With respect to applicability, concerns were frequently high, largely because the investigated populations and workflows were highly specific (single-disease cohorts, selected “clean” cases, fixed target lesions, or report-based rather than imaging-based assessment), which may limit generalizability to routine clinical practice. PROBAST appraisal suggested a high overall risk of bias across the evidence base, mainly driven by the outcome and analysis domains (see Table A3, Appendix A).

### 3.2. Report-Based Studies Outcomes

Results for report-based methods (LLM and NLP applied to radiology report text) were reported in two studies: Tan et al. and Yang et al. While both relied on report text, their evaluation frameworks differed: Tan et al. assessed response-class prediction using internal and external cohorts and primarily reported accuracy (with additional analyses of augmentation and data efficiency), whereas Yang et al. benchmarked two LLMs against expert RECIST 1.1–based annotations, reporting both accuracy and inter-rater agreement (κ), and additionally evaluated reporting time efficiency [18,19]. Notably, these performance metrics are not directly comparable across studies, as differences in cohorts, reporting conventions, reference definitions, class distributions, and evaluation protocols can markedly affect accuracy and κ. Consequently, the findings primarily inform within-study comparisons rather than cross-study head-to-head ranking.

In Tan et al., the highest performance was achieved by the transformer-based GatorTron model, which attained an accuracy of 0.8916 on the internal test set and a nearly identical accuracy of 0.8919 on an external clinical-trial cohort with RECIST-based response assessment; data augmentation yielded a small additional improvement (accuracy 0.8976). Prompt-based fine-tuning did not increase predictive accuracy, but substantially reduced the amount of training data required, maintaining good performance with as few as 500 training reports [18].

In the multicenter study by Yang et al., which enrolled a clinically homogeneous cohort of patients with nasopharyngeal carcinoma treated with induction chemotherapy followed by concurrent chemoradiotherapy and assessed at three MRI time points according to RECIST 1.1, a head-to-head comparison of two LLMs showed higher accuracy for DeepSeek-V3-0324 than for generative pre-trained transformer, GPT-4o-latest, in treatment response evaluation (TRE). DeepSeek achieved significantly higher accuracy for post-induction chemotherapy assessment (TRE-1; 96.5% vs. 82.9%, *p* < 0.001), whereas no significant differences were observed for baseline T stage (95.3% vs. 93.5%, *p* = 0.24), baseline N stage (93.8% vs. 89.6%, *p* = 0.265), or post-concurrent chemoradiotherapy assessment (TRE-2; 94.9% vs. 93.2%, *p* = 0.556). Agreement with expert annotations was consistently higher for DeepSeek (κ 0.85–0.90) than for GPT-4o (κ 0.49–0.86). In addition, across all participating radiologists, LLM assistance was associated with a significant improvement in reporting time efficiency (*p* < 0.001) [19].

### 3.3. Image-Based Studies Outcomes

Results for image-based methods were reported in two studies: Liu et al. and Dahm et al. While both implemented RECIST 1.1 from image-derived measurements, their evaluation frameworks differed. Liu et al. focused on automated diffusion-weighted imaging (DWI-based) MRI liver metastasis segmentation with automatically generated apparent diffusion coefficient maps followed by rule-based RECIST 1.1 response assignment. They reported segmentation quality using metrics such as the Dice similarity coefficient (DSC), volumetric similarity (VS), and Hausdorff distance (HD), as well as response-classification performance compared with radiologists. Dahm et al. evaluated a fully automated CT-based lesion tracking and segmentation pipeline for preselected target lesions, reporting agreement between automated and manual RECIST 1.1 measurements, as well as timepoint response classification using intraclass correlation coefficients (ICC) and kappa statistics [20,21]. Importantly, these performance metrics should not be interpreted as directly comparable across studies, because differences in cohorts, reporting conventions, reference standards, class distributions, and evaluation protocols can materially affect accuracy and κ. Accordingly, the results below primarily support within-study comparisons rather than a head-to-head ranking of report-based approaches.

In Liu et al., a 3D U-Net–based pipeline automatically segmented hepatic metastases on baseline and post-treatment liver MRI (DWI-based). A rule-based program then selected up to five measurable target lesions (longest diameter > 10 mm), computed the sum of longest diameters, incorporated new-lesion detection, and assigned RECIST 1.1 response categories. In the validation cohort, segmentation performance was DSC 0.85 ± 0.08, VS 0.89 ± 0.09, and HD 25.53 ± 12.11 mm. For treatment response assessment, classification accuracy was 0.77 for an attending radiologist (R1), 0.65 for a fellow radiologist (R2), and 0.74 for the automated segmentation-based approach; the corresponding area under the curve (AUC) values were 0.81, 0.73, and 0.83, respectively. Agreement between automated response assessment and the manual annotation–based reference standard was moderate (κ = 0.60; 95% CI 0.34–0.84) [20].

In Dahm et al., an nnU-Net–based pipeline enabled fully automated target-lesion re-identification, tracking, and segmentation across baseline and first follow-up CT in metastatic melanoma, followed by automated extraction of RECIST 1.1 diameters (long axis for non-nodal lesions; short axis for lymph nodes) and automated assignment of timepoint response categories (new lesions were not considered, and target lesions were predefined). Manual RECIST 1.1 measurements by three radiologists demonstrated excellent intra- and inter-reader reliability (ICCs > 0.90). Agreement between timepoint response derived from the mean manual diameters and the automated diameter-based assessment ranged from moderate to almost perfect (the agreement between manual diameter–based responses and automated volumetric responses was substantial (Fleiss’ κ 0.66–0.68), while the agreement between automated diameter- and volume-derived timepoint responses ranged from moderate to nearly ideal (Cohen’s κ 0.81)). Cohen’s kappa for the overall agreement was between 0.67 and 0.76 [21].

## 4. Discussion

AI is increasingly impacting oncology not only through classification models but also by transforming the entire imaging process—from acquisition and reconstruction to quantitative analysis—thus changing what can be measured and how consistently it can be measured over time [14]. AI-based workflows improve consistency, efficiency, and scalability of response assessment in routine practice and clinical trials [22]. As Smesseim et al. note, “the question remains whether AI will ultimately surpass traditional tumor measurement criteria” [23]. At the current stage of development, although AI provides promising support in image analysis, particular caution is required when integrating different data sources, such as images and report data, as their improper coordination can lead to erroneous interpretations [9]. Currently, the use of AI in RECIST-based response assessment remains a strong, yet still supportive, tool that does not replace the decisions made by the physician.

In practice, this concept is not new: since the 1990s, clinical computer-aided detection (CAD) systems have been developed as a “second reader”—for example, in mammography, where early commercial solutions received approval from the Food and Drug Administration (FDA) in 1998 to support interpretation and standardize the detection of abnormalities [24,25]. A similar assessment paradigm—comparing “without CAD” and “with CAD” radiologist results—was also applied in other modalities, such as CT colonography, where studies examined the impact of CAD as a second reader on diagnostic accuracy and variability between readers of different experience levels [26].

The application of AI to RECIST-oriented response assessment is only beginning to emerge. In our systematic review, only four eligible studies met the inclusion criteria (two image-based and two report-based), underscoring that the evidence base remains limited. Importantly, our RECIST-aligned eligibility criteria prioritized clinical interpretability and comparability with guideline-based workflows; consequently, we did not include studies evaluating exclusively volumetric or radiomics-based endpoints, which may represent a promising but methodologically distinct evidence base.

The identified report-based study by Yang et al. indicated that not all LLMs are equally suitable for RECIST-aligned treatment response assessment. The two modern LLMs were compared head-to-head against expert RECIST 1.1–based annotations in a clinically homogeneous cohort of patients with nasopharyngeal carcinoma, yet their performance differed substantially for response assessment after induction chemotherapy (TRE-1): DeepSeek-V3-0324 clearly outperformed GPT-4o-latest (96.5% vs. 82.9%, *p* < 0.001) and achieved consistently higher agreement with experts (κ 0.85–0.90 vs. 0.49–0.86), while differences for TRE-2 were not statistically significant [19]. This suggests that model choice can materially affect the reliability of RECIST-aligned classification, particularly in threshold-based criteria where correct interpretation of longitudinal comparisons, subtle report phrasing, and progression-defining cues (e.g., new lesions or non-target progression) is critical. Importantly, Yang et al. also demonstrated a practical benefit: using an LLM as a radiologist assistant was associated with significantly improved reporting time efficiency (*p* < 0.001), supporting a role for AI in augmenting—rather than replacing—expert review.

Tan et al. further show that “fitness” depends not only on architecture but also on generalizability across reporting contexts and data efficiency. The transformer-based GatorTron achieved nearly identical accuracy on an internal test set and an external clinical-trial cohort with RECIST-based assessment (0.8916 vs. 0.8919), while data augmentation yielded only modest improvement (to 0.8976). Prompt-based fine-tuning did not increase peak accuracy but improved feasibility by substantially reducing labeled-data requirements, maintaining good performance with as few as 500 training reports [18]. In this setting, the primary added value of AI is scalability—automated assignment of response categories from large volumes of report text with comparable performance across cohorts—supporting potential use for large-scale response extraction (e.g., in real-world data analyses). However, evidence remains limited on how robust report-based LLM/NLP systems are to inter-institutional variation in reporting templates, terminology, and documentation practices; therefore, performance observed in one setting may not directly translate to others without local validation or calibration. Related head-to-head benchmarks in radiology report parsing/classification (e.g., BioBERT/RadBERT variants with hospital-specific domain adaptation compared against open-source LLMs) likewise show that performance is model- and context-dependent, underscoring the need for explicit validation prior to deploying RECIST-aligned automation [12]. Taken together, these findings indicate that report-based NLP/LLM systems for RECIST-aligned response classification are not interchangeable: model choice and tuning strategy can materially affect generalizability, agreement with reference standards, and practical deployability under real-world reporting conditions.

For image-based approaches (direct analysis of raw imaging), the two studies illustrate the potential of automating key RECIST steps, but also the strong dependence on task definition and imaging/clinical conditions. In Liu et al. (DWI-based liver MRI for hepatic metastases), AI automated lesion segmentation enabling rule-based target selection and RECIST 1.1 response assignment. Despite strong segmentation metrics (DSC ~0.85), response classification performance was moderate (accuracy 0.74 for AI vs. 0.77 and 0.65 for two radiologists), with moderate agreement with the reference standard (κ ~0.60). This suggests that high segmentation quality does not automatically lead to high RECIST concordance due to error accumulation across multiple steps (target selection, sum of diameters, new lesion detection [20]). In contrast, Dahm et al. used a more controlled scenario: fully automated re-identification and tracking of predefined target lesions in metastatic melanoma between baseline and first follow-up CT, achieving excellent manual RECIST reliability (ICCs > 0.90), and moderate to nearly ideal agreement with automated response classification (κ 0.67–0.76) [21].

Key insights highlight the importance of model selection, task definition, and clinical context in AI-driven RECIST assessments. For report-based methods, models like DeepSeek-V3-0324 outperform GPT-4o-latest, showing higher accuracy and agreement with experts, but performance varies with reporting standards and data efficiency, requiring local validation. Image-based methods reveal that strong segmentation does not always lead to high RECIST agreement due to errors in target selection and lesion detection. Liu et al. showed moderate accuracy (0.74) and agreement (κ ~0.60), while Dahm et al. achieved better agreement (κ 0.67–0.76) with predefined target lesions. The results indicate that AI can enhance standardization and automation in treatment response assessment, but its generalizability depends on capturing complex RECIST components and addressing imaging variability. The impact of scanner and protocol differences was not consistently analyzed, limiting model transportability across institutions.

We distinguish two classes of limitations: intrinsic clinical constraints of RECIST (threshold-based categorization and ambiguity near cut-offs) and technical failure modes of automated pipelines (e.g., segmentation, registration, and lesion-matching errors). Technically, while AI models can achieve high performance in tasks like lesion segmentation, errors can accumulate across the multi-step pipeline, leading to only moderate agreement with expert-derived RECIST classifications [27,28]. A major challenge is the generalizability of AI systems, which are often trained on retrospective, single-center datasets with standardized protocols; performance tends to degrade when applied across different institutions or treatment contexts [29]. This is especially critical for RECIST, as even small measurement deviations near decision boundaries can shift response categories. Overall, while promising, the results are heterogeneous, and further externally validated studies are needed before AI-based RECIST assessment is ready for broad clinical use.

Key insights highlight significant challenges related to benchmarking AI in treatment response assessment according to RECIST. Many studies are conducted within the framework of retrospective datasets or single institutions with specific protocols. In the case of Liu et al. (3D U-Net-based pipeline for hepatic metastasis), the model was trained on data from a single center, which may limit its performance in other institutions with different imaging protocols, leading to issues with result transferability. A similar problem occurs in the Dahm et al. study (nnU-Net–based pipeline for metastatic melanoma), where the system operated in a controlled environment with predefined target lesions, and transferring it to other cases or protocols may affect the results. Tan et al. (transformer-based GatorTron model) also point out the transferability issue: the model achieved high accuracy on homogeneous datasets and protocols, but results may vary when applied across different institutions or clinical contexts, highlighting the need for local validation. Yang et al. (DeepSeek-V3-0324 vs. GPT-4o-latest) emphasize the variability of results depending on report templates and terminology used in different centers, which may lead to difficulties in applying the model at a broader scale.

A limitation that complicates drawing conclusions from the data provided by this systematic review is not inherent to the review itself but rather to the artificial intelligence (AI) methods used to assess treatment response according to RECIST. Liu et al. noted that despite high-quality segmentation, the AI model achieved only moderate agreement (κ ~0.60) with RECIST classifications, highlighting the challenges in correcting measurement errors within the multi-step process. Similarly, in Dahm et al., although the agreement was good (κ 0.67–0.76), the lack of full representation of actual disease progression limited the transferability of the results. Tan et al. demonstrated that while the GatorTron model achieved high accuracy (0.8916), it was not tested across different protocols, which makes it difficult to generalize the results to other institutions. In Yang et al., variability in report templates and terminology complicated the adaptation of NLP models, further underscoring the issue of result transferability.

These findings provide clear examples of the limitations of AI in RECIST-based assessment. Previous studies (e.g., Reichenpfader et al.) have also shown that, despite high-quality segmentation, AI models only achieve moderate agreement with RECIST classifications, primarily due to the difficulties in correcting measurement errors in the multi-step process [30]. Similarly, Zech et al. found that the transferability of deep learning models across hospital systems was limited, as performance was significantly lower on external datasets than on training data [29]. This was largely due to differences in documentation practices and terminology between institutions, which affect the ability of AI models to reliably transfer between environments.

Moreover, deep learning–based imaging models are vulnerable to adversarial perturbations, such as one-pixel attacks, which can alter predictions with minimal, visually imperceptible changes, raising concerns about robustness and safety in automated RECIST assessment [31]. For oncologists, AI-generated RECIST assessments should be interpreted as decision-support tools rather than definitive response determinations, with treatment decisions remaining grounded in integrated clinical, radiologic, and temporal evaluation.

The weaknesses of our review include the limited number of available publications and the challenges in assessing their methodological quality, which may reduce the representativeness of the evidence base and limit the generalizability of our conclusions. QUADAS-2, developed for traditional diagnostic accuracy studies, does not fully address AI-specific risks such as data leakage, iterative model/prompt optimization, domain shift, or error propagation in multi-step pipelines. This is particularly relevant in Yang, where iterative prompt refinement and a RAG-based workflow may introduce overfitting or site-specific tailoring that QUADAS-2 cannot adequately evaluate. In Tan, QUADAS-2 treats curated report-based labels as the reference standard, potentially inflating performance by conflating clinical validity with documentation outcomes. In Liu, QUADAS-2 does not explicitly account for segmentation errors that may propagate to RECIST response classification, and in Dahm, it does not penalize task simplifications (e.g., fixed target lesions and omission of new lesions) that may limit generalizability to routine clinical practice. To partially address these limitations, we complemented QUADAS-2 with PROBAST, which is tailored to prediction-model studies and explicitly considers key sources of bias in the outcome and analysis domains (e.g., incorporation bias, optimism/overfitting, class imbalance, and inadequate external validation). PROBAST appraisal corroborated substantial methodological limitations across the included studies, particularly in the outcome and analysis domains, supporting a cautious interpretation of the reported performance estimates. However, even PROBAST may not fully capture deployment-specific risks of rapidly evolving AI systems (e.g., temporal drift, site-to-site variation in reporting practices, and dynamic model updates), underscoring the need for more AI-specific appraisal frameworks and better reporting standards in future research.

Finally, for rapidly evolving LLM systems, the evidence base is prone to publication bias (selective reporting of favorable results) and rapid obsolescence as model versions, prompting strategies, and deployment settings change faster than the publication cycle. Consequently, reported performance should be interpreted as time- and version-specific, and future studies should report model identifiers (version/date), inference settings, and provide periodic re-benchmarking on shared test sets.

In addition, benchmarking remains a major challenge in this field, mainly due to the lack of standardized datasets with consistent RECIST 1.1 annotations and the predominance of retrospective, single-center cohorts. In addition, ground-truth labeling varies across centers (reader workflow/adjudication, reference standard, target lesion selection, imaging protocols), limiting comparability and generalizability of reported performance.

It should be noted that AI tools for RECIST assessment will typically fall under software as a medical device regulation, requiring evidence of analytical validity, clinical performance, quality management, and post-market surveillance [32]. In the United States and the EU, additional challenges include regulatory pathways for controlled model updates (e.g., FDA approaches such as a predefined change control plan) and alignment with EU medical device requirements (CE marking under MDR/IVDR) and, where applicable, the EU Artificial Intelligence Act [33,34]. Furthermore, operational integration depends on seamless interoperability with existing clinical systems, such as PACS, RIS, and EHR, as well as robust version control and post-deployment monitoring to manage performance drift, particularly for rapidly evolving LLM-based systems [35,36].

The results presented in our review, based on the four included studies, indicate that while AI holds promising potential for treatment response assessment, the current state of the technology requires further development [37,38]. These studies, while providing valuable insights, are heterogeneous, and their number is too small to draw definitive conclusions about the readiness of AI tools for widespread clinical application. Gathering the necessary evidence for regulatory approval requires a larger number of observations and results from advanced-stage clinical trials aimed at validating the effectiveness and safety of AI tools in broader patient populations [39]. For oncologists, AI-generated RECIST assessments should be interpreted as decision-support tools rather than definitive response determinations, with treatment decisions remaining grounded in integrated clinical, radiologic, and temporal evaluation.

## 5. Conclusions

AI for RECIST-oriented treatment-response assessment appears promising but remains in an early stage. Only four studies met the inclusion criteria (two image-based and two report-based), and evidence was limited and heterogeneous, precluding meaningful pooling and constraining generalizability. Report-based methods suggest that LLMs can infer RECIST-aligned response categories from radiology report text and may improve efficiency, whereas image-based pipelines demonstrate the feasibility of deriving RECIST measurements directly from imaging, with agreement depending on the pipeline’s scope and inclusion of challenging RECIST components (e.g., new lesions and non-target disease). Overall, methodological quality was limited (QUADAS-2), with recurring concerns regarding selection/applicability and incomplete reporting in key domains. At present, the evidence supports only proof-of-concept use in controlled or research settings, rather than near-term routine clinical deployment. Therefore, AI should currently be viewed as decision support, and routine implementation should await prospective, multi-center validation using standardized benchmarks and externally locked evaluations with clearly defined RECIST 1.1 reference standards. Specifically, future benchmarks should use prospectively collected multi-center datasets with locked test splits, RECIST 1.1 reference established via blinded independent central review (with adjudication), and protocol/site-stratified reporting of agreement (e.g., κ and category-level concordance for CR/PR/SD/PD, including explicit handling of non-target disease and new lesions).

## Figures and Tables

**Table 1:** Concise comparison of AI approaches for RECIST-based treatment response.

	Image-Based Approach	Report-Based Approach
Advantages	Direct access to the primary data enables quantitative, lesion-level assessment aligned with RECIST measurements (e.g., longest diameters, sum of diameters) and supports richer imaging biomarkers beyond text (e.g., volumetry, radiomics). Automation can reduce inter-reader variability and improve measurement consistency across timepoints when robustly validated.	Highly scalable in routine-care and multi-center settings because reports are widely available and easier to handle than imaging data. Enables rapid extraction of response/progression signals and longitudinal status from real-world documentation without requiring image segmentation.
Limitations	Requires large, well-annotated imaging datasets (often lesion-level labels/segmentations and longitudinal matching), which are costly and time-consuming to create. Performance may degrade under domain shift due to heterogeneity in scanners, acquisition protocols, reconstruction settings, and contrast phases; integration with clinical/PACS workflows and quality control adds operational complexity.	Constrained to what is documented in the report; key RECIST details (target lesion selection and complete, reproducible measurements) may be absent or inconsistently reported, limiting formal RECIST fidelity. Susceptible to variability in reporting style, templates, terminology, and language, and inherits radiologist interpretation bias and label noise from non-standardized narratives.

PACS: Picture Archiving and Communication System; RECIST: Response Evaluation Criteria in Solid Tumors.

**Table 3:** Standardized summary of evaluation level and metrics.

Study	Input Type	Evaluation Level	Primary Metrics	Reference Standard
Tan et al. (2023) [18]	Report text	Response classification	Accuracy (primary); κ NR	Adjudicated RECIST 1.1 (trial cohort)
Yang et al. (2025) [19]	Report text	Response classification; workflow impact	Accuracy; κ; reporting time	Expert RECIST 1.1–based annotations
Liu et al. (2022) [20]	Raw imaging (MRI)	Segmentation/measurement; response classification	DSC; VS; HD; Accuracy; AUC; κ	Manual radiologist-derived reference
Dahm et al. (2024) [21]	Raw imaging (CT)	Measurement agreement; response agreement	ICC; κ	Manual RECIST 1.1 by radiologists

AUC: area under the curve; CT: computed tomography; DSC: Dice similarity coefficient; HD: Hausdorff distance; ICC: intraclass correlation coefficients; MRI: magnetic resonance imaging; NR: not reported; RECIST: Response Evaluation Criteria in Solid Tumors; VS: volumetric similarity.

## Data Availability

The original contributions presented in this study are included in the article. Further inquiries can be directed to the corresponding author.

## Abbreviations

The following abbreviations are used in this manuscript:

AI	artificial intelligence
AUC	area under the curve
Bi-LSTM	bidirectional long short-term memory
CAD	computer-aided detection
CNN	convolutional neural network
CR	complete response
CT	computed tomography
DL	deep learning
DSC	dice similarity coefficient
DWI	diffusion-weighted imaging
FDA	Food and Drug Administration
GPT	Generative Pre-trained Transformer
HD	Hausdorff distance
ICC	intraclass correlation coefficients
LLM	large language models
MeSH	Medical Subject Headings
ML	machine learning
MM	metastatic melanoma
MRI	magnetic resonance imaging
NED	no evidence of disease
NLP	natural language processing
NPC	nasopharyngeal carcinoma
NR	not reported
PACS	Picture Archiving and Communication System
PD	progressive disease
PR	partial response
PRISMA	Preferred Reporting Items for Systematic Reviews and Meta-Analyses
QUADAS-2	Quality Assessment of Diagnostic Accuracy Studies 2
RAG	retrieval-augmented generation;
RECIST	Response Evaluation Criteria in Solid Tumors
SD	stable disease
TRE	treatment response evaluation
VS	volumetric similarity

## Appendix A

**Table A1:** Search strings used across databases for systematic literature review.

Database	Search String
Embase	(‘artificial intelligence’/exp OR ‘machine learning’/exp OR ‘deep learning’/exp OR ‘neural network’/exp OR ai OR “artificial intelligence” OR “machine learning” OR “deep learning” OR neural network*) AND (‘solid tumor’/exp OR ‘neoplasm’/exp OR ‘cancer’/exp OR solid tumor* OR tumor* OR neoplasm* OR cancer) AND (‘response evaluation criteria in solid tumors’/exp OR recist OR “response evaluation criteria in solid tumors”). Limits: [embase]/lim, [humans]/lim, [english]/lim, (‘article’/it OR ‘review’/it), year ≥ 2015.
Medline	(Artificial Intelligence OR Machine Learning OR Deep Learning OR Neural Networks).mp. AND (Radiology OR Neoplasms OR Oncology OR Solid Tumors OR Cancer).mp. AND (Treatment Outcome OR Tumor Response OR Radiological Response OR Response Evaluation Criteria in Solid Tumors OR RECIST).mp. Limits: English, Humans, 2015–current; preprints excluded.
Scopus	(TITLE-ABS-KEY ((‘artificial intelligence’ OR ‘machine learning’ OR ‘neural network’)) AND TITLE-ABS-KEY ((‘solid tumor’ OR ‘neoplasm’ OR ‘cancer’)) AND TITLE-ABS-KEY ((‘RECIST’ OR ‘response evaluation criteria in solid tumors’))) AND (LIMIT-TO (LANGUAGE, “English”))
Web of Science	TS = (“artificial intelligence” OR “machine learning” OR “deep learning” OR “neural network”) AND TS = (“solid tumor” OR “neoplasm” OR “cancer”) AND TS = (“RECIST” OR “response evaluation criteria in solid tumors”) Index Date: 1 January 2015 to 6 December 2025
Cochrane Library	((MeSH descriptor: [Artificial Intelligence] explode all trees) OR (MeSH descriptor: [Machine Learning] explode all trees) OR (artificial intelligence or AI or “machine learning” or “deep learning” or neural network* or convolutional neural network* or CNN or transformer* or “computer vision” or radiomic* or “image analysis” or “image segmentation” or segment* or automat* or algorithm*):ti,ab,kw) AND (RECIST or “Response Evaluation Criteria in Solid Tumors” or “Response Evaluation Criteria in Solid Tumours”):ti,ab,kw AND ((MeSH descriptor: [Diagnostic Imaging] explode all trees) OR (MeSH descriptor: [Tomography, X-Ray Computed] explode all trees) OR (MeSH descriptor: [Magnetic Resonance Imaging] explode all trees) OR (MeSH descriptor: [Positron-Emission Tomography] explode all trees) OR (radiolog* or imaging or image* or “computed tomography” or CT or MRI or “magnetic resonance” or PET or “positron emission” or ultrasonograph* or “response assessment” or “response evaluation” or “treatment response” or “tumor response” or “tumour response” or “radiologic response” or “radiological response” or “imaging response” or lesion measur* or tumor measur* or tumour measur* or volumetr*):ti,ab,kw) AND ((MeSH descriptor: [Neoplasms] explode all trees) OR (cancer* or neoplasm* or tumour* or tumor* or carcinoma* or sarcoma* or melanoma* or (solid NEXT tumor*) or (solid NEXT tumour*)):ti,ab,kw)

**Table A2:** Quality assessment of included publications with QUADAS-2.

Publication	Risk of Bias				Applicability		
	D1	D2	D3	D4	D1	D2	D3
Tan et al. (2023) [18]	High	Low	Low/Unclear	Low/Unclear	High	Moderate	High
Yang et al. (2025) [19]	Unclear	High	Low/Unclear	Unclear	High	High	Moderate
Liu et al. (2022) [20]	High	Unclear	Unclear/High	Low/Unclear	High	High	Moderate/High
Dahm et al. (2024) [21]	Low	Unclear	Low	Unclear	High	High	High

D1—patient selection; D2—index test (AI); D3—reference standard; D4—flow and timing.

**Table A3:** Risk of bias and applicability assessment of included prediction model studies using the PROBAST tool.

Publication	Participants	Predictors	Outcome	Analysis	Overall	Overall
	Risk of Bias/Applicability			Risk of Bias	Risk of Bias	Applicability
Tan et al. (2023) [18]	High/High	Low/Unclear	Low/Unclear	Unclear	High/Unclear	High
Yang et al. (2025) [19]	Unclear/High	Low/High	High/High	High	High	High
Liu et al. (2022) [20]	High/Unclear	Unclear/Unclear	High/Unclear	High	High	Unclear/High
Dahm et al. (2024) [21]	High/High	High/High	Unclear/High	Unclear/High	High	High
